# Lord Onslow and Local Committees

**Published:** 1921-09-03

**Authors:** 


### The Hospital

The Workers' Newspaper of Administrative Medicine and Institutional Life, Administration, National Insurance and Health.

No. 1839, Vol. LXX. SATURDAY, SEPTEMBER 3, 1921. PRICE TWOPENCE

### LORD ONSLOW AND LOCAL COMMITTEES.

On another page we give the substance of an interview which the Earl of Onslow, Chairman of the Hospitals Commission, has granted to a provincial newspaper upon the task of the Commission and the functions of the local committees on whose advice it will largely rely before making grants. Lord Onslow was at pains to show that the new local committees, to be formed in each county, will be advisory bodies, and not the masters of the hospitals within their area. He also urged the solvent hospitals to co-operate with the less prosperous, because only by such co-operation can the voluntary system be saved. We confess that these two statements seem to us to imply a slight confusion of ideas. It is because the individual hospitals have not yet achieved anything in the way of co-operation that more or less independent bodies were recommended by the Cave Committee, which feels that only by such independent pressure from outside can any real co-operation be accomplished. The local committees were then armed with the power of the purse, since requests for grants must come through them, in order that their views might be respected. On the other hand, we appreciate Lord Onslow's desire to discount hostility to the local committees, but his attempt to show that the hospitals have nothing to fear from the local committees may induce the more independent hospitals to feel that they need not pay too much attention to the committees' advice. If the local committees are as innocuous as Lord Onslow seems to imply, how can they help the cause of co-operation? If, on the other hand, the committees can secure a greater amount of co-operation than the separate hospitals would themselves create, then, obviously, the committees must be able to bring some pressure to bear. Since, on the whole, we believe that the independence of the separate hospitals needs correction, we are not afraid of advisory bodies which can make their advice a thing to be reckoned with.

Doubtless, however, some unreasonable alarm has been aroused. Indeed, this is evident from a recent speech by Mr. J. W. Willis-Bund, who presided over a conference of hospital representatives at Worcester, which was held to discuss the formation of a local committee for the county. Mr. Willis-Bund said that the meeting was the first important step toward making more hospitals State institutions, paid for out of public funds. That, of course, was a reckless statement of opinion, for anyone who has read at all carefully the Report of the Cave Committee, and the statement issued by the Hospitals Commission, must see that the appointment of local committees is a step toward co-ordination and nothing more. At the same meeting Mr. Geoffrey New, of Evesham, gave a much more correct statement of the facts, but we emphasise Mr. Willis-Bund's expressions because they show the state of opinion with which Lord Onslow has to deal, and the misunderstandings which the Commission already excites.

There remains the position of the local committees themselves, though for the most part these are only in process of formation. Our chief fear in regard to them is that the amount of money which they will be able to recommend may not be large enough to win serious attention for their other recommendations. Half-a-million between the hospitals of London and the provinces is a small sum, comparatively. The paragraphs devoted to the committees in the Report of the Cave Committee show that these bodies were intended to have at least as much control over hospitals as King Edward's Fund exercises in London. They were described as independent bodies, and were made independent because experience has shown that, left to themselves, the voluntary hospitals are too jealous of their independence, and too parochial in their views, to co-operate effectively. It remains to be seen how far the remedy proposed will be sufficient; for no impartial opinion can deny that a remedy is required. Unfortunately, at the very start the local committees were disarmed of half their powers by the act of the Government in cutting down the proposed grant by half.

It must not be thought from these remarks that we are placing all our faith on new and untried bodies. No one can tell at present how effective or ineffective the new committees will be. Our point merely is that for hospital co-operation, which is very badly needed, a new instrument has been devised; and since the end is a wise one, the instrument should be respected, and encouraged to make the best of itself. If there had been any real solidarity and vision, the hospitals would have formed the new instrument themselves. As they did not do so, it has had to be made from without; and we think that a lamentable short-sightedness will be shewn if the hospitals do not do their best to work well with the new bodies. It may very well prove true that the voluntary *system* depends upon them. Hitherto hospitals have talked much of the system, but thought only of their own needs and affairs. Success will mean the sacrifice of selfishly narrow views, but it is these narrow views, and not the new control imposed upon them, which is the force to be dreaded.

